# Impact of mixed meal tolerance test composition on measures of beta-cell function in type 2 diabetes

**DOI:** 10.1186/s12986-021-00556-1

**Published:** 2021-05-04

**Authors:** Theresa Kössler, Pavel Bobrov, Klaus Strassburger, Oliver Kuss, Oana-Patricia Zaharia, Yanislava Karusheva, Clara Möser, Kálmán Bódis, Volker Burkart, Michael Roden, Julia Szendroedi, for the GDS Group, M. Roden, H. Al-Hasani, B. Belgardt, V. Burkart, A. E. Buyken, G. Geerling, C. Herder, J. H. Hwang, A. Icks, K. Jandeleit-Dahm, S. Kahl, J. Kotzka, O. Kuß, E. Lammert, W. Rathmann, J. Szendroedi, S. Trenkamp, D. Ziegler

**Affiliations:** 1 Division of Endocrinology and Diabetology, Medical Faculty, Heinrich Heine University, Moorenstr. 5, 40225 Düsseldorf, Germany; 2 Institute for Clinical Diabetology, German Diabetes Center, Leibniz Center for Diabetes Research At Heinrich Heine University Düsseldorf, Düsseldorf, Germany; 3 German Center for Diabetes Research (DZD), München-Neuherberg, Germany; 4 Institute for Biometrics and Epidemiology, German Diabetes Center, Leibniz Center for Diabetes Research at Heinrich Heine University Düsseldorf, Düsseldorf, Germany; 5 Department of Internal Medicine I and Clinical Chemistry, University Hospital Heidelberg, Heidelberg, Germany

**Keywords:** Mixed meal tolerance test, Type 2 diabetes, Intra-individual variation, Inter-individual variation, Beta-cell function

## Abstract

**Background:**

Application of mixed meal tolerance tests (MMTT) to measure beta-cell function in long-term studies is limited by modification of the commercial products occurring over time. This study assessed the intra-individual reliability of MMTTs and compared the effects of liquid meals differing in macronutrient composition on the estimation of beta-cell function in type 2 diabetes (T2DM).

**Methods:**

To test the reliability of MMTTs, 10 people with T2DM (age 58 ± 11 years, body mass index 30.0 ± 4.9 kg/m^2^) received *Boost®* *high Protein 20 g protein* three times. For comparing different meals, another 10 persons with T2DM (58 ± 5 years, 31.9 ± 5.3 kg/m^2^) ingested either *Boost®* *high Protein 20 g protein* or the isocaloric *Boost®* *high Protein 15 g protein* containing 35% less protein and 18% more carbohydrates. C-peptide, insulin and glucose release were assessed from the incremental area under the concentration time curve (iAUC) and the intra- and inter-individual variation of these parameters from the coefficients of variations (CV).

**Results:**

Repetitive ingestion of one meal revealed intra-individual CVs for the iAUCs of C-peptide, insulin and glucose, which were at least 3-times lower than the inter-individual variation of these parameters (18.2%, 19.7% and 18.9% vs. 74.2%, 70.5% and 207.7%) indicating a good reliability. Ingestion of two different meals resulted in comparable intra-individual CVs of the iAUCs of C-peptide and insulin (16.9%, 20.5%).

**Conclusion:**

MMTTs provide reliable estimation of beta-cell function in people with T2DM. Furthermore, moderate differences in the protein and carbohydrate contents in a standardized liquid meal do not result in relevant changes of C-peptide and insulin responses.

*Trial registration*: Clinicaltrials.gov, Identifier number: NCT01055093. Registered 22 January 2010 – Retrospectively registered, https://www.clinicaltrials.gov/ct2/show/study/NCT01055093

## Background

The mixed meal tolerance test (MMTT), based on the ingestion of a standardized liquid meal, is commonly used to assess pancreatic beta-cell function in people with diabetes [1, 2]. Further widely applied methods for measuring beta-cell function are the intravenous and the oral glucose tolerance test (OGTT), the hyperinsulinemic-hyperglycemic clamp test, and the glucagon stimulation test [3]. However, only MMTT and OGTT can trigger incretin secretion and therefore provoke a physiological beta-cell response compared to the other methods. Moreover, the secretion of incretins is modulated not only by ingestion of glucose, but also by other nutrients like proteins and fat, which are only provided by the MMTT [3–5]. Comparative studies have shown that MMTT induces a stronger beta-cell response than the OGTT [4], comes with the additional benefit of easy administration and is a suitable method to assess physiological beta-cell function in long-term cohorts [3, 4]. Furthermore, the MMTT represents a reliable and reproducible method of measuring parameters of beta-cell function in cohorts across a wide spectrum of disorders of glucose homeostasis [3, 6, 7]. However, the modification of recipes in commercially available liquid meals may cause deviations of beta-cell response, which limit the application in long-term studies. Different macronutrient composition as well as individual physiological variation are known to influence the postprandial metabolic response both within and between individuals, which can make it difficult to compare the results in large cohorts [8–10]. In order to address the robustness of using MMTTs, the present study aims to assess the intra-individual reliability of MMTT in a defined population of people with type 2 diabetes (T2DM). Additionally, the study aims to test whether the use of two liquid meals with identical amounts of fat and mono- and disaccharides but different protein and carbohydrate content affects the estimation of pancreatic beta-cell function in T2DM.

## Methods

### Participants and study design

The analyses included people with T2DM from the German Diabetes Study (GDS; clinicaltrials.gov: NCT01055093) [11] with a known diabetes duration of < 6 years. Participants were treated with lifestyle modification and/or oral glucose lowering medication and gave their informed consent to the study protocol, which was approved by the Ethics Board of the Medical Faculty of the Heinrich-Heine-University Düsseldorf.

### Mixed meal tolerance test

Each MMTT was performed with commercial standardized liquid meals after 10-h overnight fasting and after participants had stopped oral glucose-lowering medication for at least 3 days. For testing the intra-individual reliability of MMTT, 10 individuals with T2DM were given 378 g of *Boost®* *20 g protein* (365.8 kcal, 9.1 g fat, 42.5 g carbohydrates (22.8 g mono- and disaccharides) and 30.7 g protein; Nestlé Health Care Nutrition) three times under identical conditions within one month. For comparing the effects of meals with different macronutrient composition, two MMTTs were performed in a cross-over design in ten other people with T2DM. In a randomized order, the study participants received 378 g of either *Boost®* *20 g protein* or *Boost®* *15 g protein* (365.8 kcal, 9.1 g fat, 50.1 g carbohydrates (22.8 g mono- and disaccharides) and 22.8 g protein; Nestlé Health Care Nutrition), separated by a washout period of 1–2 weeks. Blood samples were obtained before (-1 min) and 15, 30, 60, 90, 120 and 180 min after meal ingestion to measure parameters of beta-cell function.

### Statistical analyses

The incremental area under the concentration time curve (iAUC) was calculated for parameters of pancreatic beta-cell function using the trapezoidal method [12]. Paired t-tests were used for comparisons between iAUCs obtained from different meals. The intra-individual variation of the iAUCs was calculated using the coefficient of variation (CV = 100 × standard derivation (SD)/mean). The mean obtained for each study participant was used to calculate the inter-individual CVs and reflects the variability between participants. The intraclass correlation coefficient (ICC) was used to quantify the relation between intra- and inter-individual variability and thus provides information about the reliability of MMTT. The ICC was calculated as (mean squared between individuals—mean squared within individuals)/[mean squared between individuals + (number of observations − 1) × mean squared within individuals)] [13]. The ICC can vary between 0 and 1, with a value closer to 1 indicating lower variability within than between persons and therefore a good reliability [9]. Statistical analyses were performed with SAS (SAS ®, Cary, NC, USA) PROC MIXED, Version 9.3.

## Results

### Anthropometry

Individuals with T2DM ingesting either one meal three times or two different meals had similar age and body mass index (BMI) (30% female, age 58 ± 11 years, BMI 30.0 ± 4.9 kg/m^2^ vs. 40% female, 58 ± 5 years, 31.9 ± 5.3 kg/m^2^). All study participants had excellent glucometabolic control (HbA1c 6.3 ± 0.6% vs. 6.5 ± 0.7%) (Table 1).

**Table 1:** Baseline characteristics of the study participants

Variables	MMTT-comparison of one meal three times	MMTT-comparison of two different meals
N (% male)	10 (70)	10 (60)
Age [years]	58 ± 11	58 ± 5
BMI [kg/m^2^]	30.0 ± 4.9	31.9 ± 5.3
Disease duration since diagnosis [months]	22.7 ± 27.8	31.1 ± 21.3
HbA1c [%]	6.3 ± 0.6	6.5 ± 0.7
HbA1c [mmol/mol]	45 ± 6	48 ± 8
Oral antidiabetic treatment, N	7/ 10	7/ 10

Data are n (%) or mean ± SD

### Metabolic response to standardized meals

**Fig. 1:**
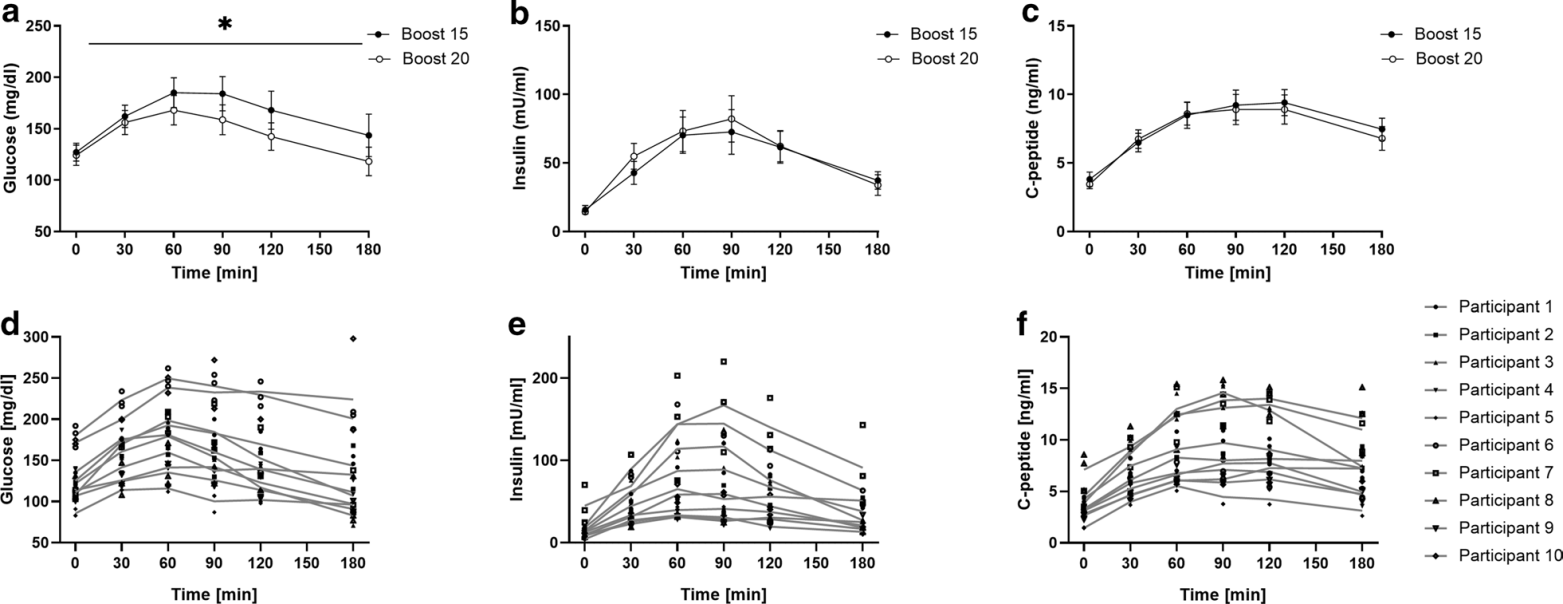
Metabolic response to mixed meal tests. Means and standard deviation for glucose (**a**), insulin (**b**) and C-peptide (**c**) when ingesting two different mixed meal products (Boost 15 and Boost 20). Individual measurements for each participant and the calculated average postprandial excursions for glucose (**d**), insulin (**e**) and C-peptide (**f**) when ingesting the same mixed meal product three times. *p < 0.05 comparing iAUCs using paired t-test

We recorded glucose, insulin and C-peptide after ingesting either one meal three times or two meals with different composition. Comparing the iAUC for glucose, C-peptide and insulin between two different meals showed similar insulin and C-peptide excursions, but higher glucose excursions between meals (*p* < 0.05; Fig. 1).

### Intra- and inter-individual variation of measures of beta-cell function

Table 2 shows the CVs for the iAUCs of parameters characterizing glucose metabolism. When testing one meal three times, the intra-individual variations for C-peptide, insulin and glucose were within a low range between 18.2% and 19.7%. This was also reflected by ICC (0.69, 0.70 and 0.89) indicating moderate to strong reliability. The inter-individual CV was at least three times higher compared to the intra-individual CV of these parameters.

**Table 2:** Intra-and inter-individual variation of parameters of glucose metabolism during two different MMTTs

Variables	MMTT-comparison of one meal three times				MMTT-comparison of two different meals			
	iAUC	CV Intra-individual variation (%)	CV Inter-individual variation (%)	ICC	iAUC	CV Intra-individual variation (%)	CV Inter-individual variation (%)	ICC
C-peptide	672 (276; 1269)	18.2 (2.6; 40.3)	74.2	0.69	759 (377; 1339)	16.9 (0.6; 77.9)	42.7 (41.6; 43.9)	0.51
Insulin	5456^†^ (2363; 9281)	19.7^†^ (2.2; 52.4)	70.5^†^	0.70	7082^§^ (2473; 17,080)	20.5^§^ (5.2; 61.8)	69.0^§^ (64.6; 73.4)	0.59
Glucose	4435 (− 1706; 12,671)	18.9 (− 80.7; 74.9)	207.7	0.89	5276 (810; 15,270)	45.9 (6.9; 97.2)	69.1 (66.6; 71.6)	0.53

Data are means, coefficient of variation (CV), minimum and maximum and intraclass correlation coefficient (ICC). Data are given in ng*ml^−1^ *3 h^−1^ for iAUC C-peptide, mU*l^−1^*3 h^−1^for iAUC insulin, mg*dl^−1^*3 h^−1^ for iAUC glucose

Data are available for ^§^n = 17 out of 20 measurements or ^†^n = 27 out of 30 measurements

iAUC, incremental area under the curve; CV, coefficient of variation; ICC, intraclass correlation coefficient; MMTT, mixed meal tolerance test

Ingestion of two meals different in protein and carbohydrate content results in comparable intra-individual CVs for C-peptide and insulin (16.9 and 20.5%) while the intra-individual variation of glucose was about two times higher. However, the inter-individual variation of C-peptide, insulin and glucose was up to threefold higher ranging from 42.7 to 69.1%. The ICC of the three parameters was between 0.51 and 0.59.

## Discussion

This study shows that the MMTT represents a reliable method for the estimation of beta-cell function in people with T2DM. Furthermore, a moderate change in protein and carbohydrate content with a constant amount of fat and available mono- and disaccharides may have no relevant effect on central parameters of pancreatic beta-cell function.

C-peptide, insulin and glucose levels show similar intra-individual variation, when testing one commercial liquid meal in the same person three times. The inter-individual variation of these parameters were at least threefold higher than the intra-individual variation, indicating good reliability. The ICCs of C-peptide, insulin and glucose were also in a range indicating moderate to high reliability [3, 9], with the best reliability for glucose, in line with a previous study (ICC = 0.83) [6]. However, that study only included metabolically healthy men. Another study showed ICC ranging from 0.3 to 0.8 depending on included participants and assessed parameters in MMTT [3], which might be explained by the application of an MMTT-protocol containing both solid and liquid components. Nevertheless, these studies confirm our findings that the MMTT represents a good method for the reliable measurement of beta-cell function.

The present study is in agreement with a previous study examining metabolic responses to standardized meals [14]. The postprandial variations, as assessed from ICC, for glucose and C-peptide were 0.74 and 0.62 compared to 0.89 and 0.69 in our study, respectively. Additionally, inter-individual variation also showed differences even between identical twins, attributable to modifiable factors. These include baseline values, habitual diet, genetic factors, meal timing and meal composition [14]. Of note, the previous study included only healthy individuals, specifically excluding participants taking medication for T2DM, and used an OGTT or solid-food based meals for the examinations. The present study expands on these observations by including participants with T2DM and using standardized liquid meals to assess postprandial glycemic and beta-cell responses.

Our study also shows that changes in protein content of < 35% and carbohydrate content of < 18%, do not result in relevant changes of postprandial C-peptide or insulin responses as central parameters for the characterization of beta-cell function, provided that the amounts of fat and available mono- and disaccharides remain identical. However, the intra-individual variation of postprandial glucose was at least two times higher compared to postprandial C-peptide and insulin response. Furthermore, the variance between postprandial responses may mainly result from differences in macronutrient composition, individual glucose absorption rates and meal specific metabolic response [14]. According to previous studies, examining the impact of solid standardized meals in metabolically healthy persons, a moderate change in macronutrients does not alter postprandial insulin or glucose responses [10, 15]. However, these observations are difficult to compare with results obtained with liquid meals due to different macronutrient composition, food processing and consequent availability of nutrients, as well as individual chewing and digestive efficiency.

Furthermore, the ICC indicates that the variability of postprandial C-peptide, insulin and glucose within a person is comparable to the variability between the individuals in this cohort. Remarkably, other studies observed larger variability within than between the individuals when comparing repeatedly performed test meals [16–18]. However, the majority of these studies assessed the variability of glycemic-index values and thereby focused on the impact of dietary carbohydrates [9, 17, 18].

The comparison of the two liquid meals shows at least twofold larger intra-individual variation of glucose compared to the intra-individual variation of C-peptide and insulin. While the amount of available mono- and disaccharides were identical, the differences in total carbohydrate affected the excursions of plasma glucose following ingestion of a liquid meal. Not surprisingly, meals with different carbohydrate content resulted in different glucose excursions. However, these differences did not affect measures of beta cell function as C-peptide and insulin excursions were not different between meals. Thus, while beta-cell function is similar, glucose levels might not yield comparable data when assessed from meals varying moderately in protein and carbohydrate content despite identical mono- and disaccharide contents. Accordingly, the macronutrient composition, within the range tested in this study, did not affect the central measures of beta-cell function.

The strengths of the study lie in the repeated MMTT testing, three times, under standardized conditions of comprehensively phenotyped people with T2DM, in line with studies recommending to repeat tests at least once to account for the variation within individuals. Furthermore, the test meal used is a standardized commercial liquid meal with a balanced macronutrient composition to minimize variability due to composition, preparation or other individual variations for example in chewing efficiency of a solid meal.

Limitations are the small size of study participants in each group, due to the limited availability of one commercial liquid meal. However, participants showed a similarly good glycemic control and comparable anthropometric characteristics. To reduce intra-individual variations the MMTTs were performed under identical conditions and a crossover design was chosen when comparing different meals.

## Conclusion

Taken together, the MMTT is a reliable method for assessing beta-cell function in people with T2DM. Furthermore, moderate changes in protein and carbohydrate content with identical amounts of available mono- and disaccharides do not seem to cause statistical and clinically relevant changes in post-prandial C-peptide and insulin response. However, glucose excursions following meal intake differed, which may suggest that markers of glucose uptake should not be compared between MMTTs with differences in macronutrient composition.

## Data Availability

A request and transfer process has been established so that researchers may apply for data by contacting the study coordinators via email (GDS@ddz.de). Once approved by the steering committee, the requesting researcher and the principal investigator of GDS sign a contract on the terms and conditions of data transfer and transmission of results back to the German Diabetes Center.

## Abbreviations

AUC: Area under the curve
CV: Coefficient of variation
FFA: Free fatty acids
ICC: Intraclass correlation coefficient
MMTT: Mixed meal tolerance test
OGTT: Oral glucose tolerance test
T2DM: Type 2 diabetes mellitus

## References

[1] Besser REJ, Shields BM, Casas R, Hattersley AT, Ludvigsson J (2013). Lessons from the mixed-meal tolerance test: use of 90-minute and fasting C-peptide in pediatric diabetes. Diabetes Care.

[2] Greenbaum CJ, Mandrup-Poulsen T, McGee PF, Battelino T, Haastert B, Ludvigsson J (2008). Mixed-meal tolerance test versus glucagon stimulation test for the assessment of beta-cell function in therapeutic trials in type 1 diabetes. Diabetes Care.

[3] Shankar SS, Vella A, Raymond RH, Staten MA, Calle RA, Bergman RN (2016). Standardized mixed-meal tolerance and arginine stimulation tests provide reproducible and complementary measures of β-cell function: results from the foundation for the National Institutes of Health Biomarkers Consortium Investigative Series. Diabetes Care.

[4] Rijkelijkhuizen JM, Girman CJ, Mari A, Alssema M, Rhodes T, Nijpels G (2009). Classical and model-based estimates of beta-cell function during a mixed meal vs. an OGTT in a population-based cohort. Diabetes Res Clin Pract..

[5] Ahrén B (2013). Incretin dysfunction in type 2 diabetes: clinical impact and future perspectives. Diabetes Metab.

[6] Paglialunga S, Guerrero A, Roessig JM, Rubin P, Dehn CA (2016). Adding to the spectrum of insulin sensitive populations for mixed meal tolerance test glucose reliability assessment. J Diabetes Metab Disord.

[7] Guglielmi C, Del Toro R, Lauria A, Maurizi AR, Fallucca S, Cappelli A (2017). Effect of GLP-1 and GIP on C-peptide secretion after glucagon or mixed meal tests: Significance in assessing B-cell function in diabetes. Diabetes Metab Res Rev.

[8] Brodovicz KG, Girman CJ, Simonis-Bik AMC, Rijkelijkhuizen JM, Zelis M, Bunck MC (2011). Postprandial metabolic responses to mixed versus liquid meal tests in healthy men and men with type 2 diabetes. Diabetes Res Clin Pract.

[9] Williams SM, Venn BJ, Perry T, Brown R, Wallace A, Mann JI (2008). Another approach to estimating the reliability of glycaemic index. Br J Nutr.

[10] Nuttall FQ, Gannon MC, Wald JL, Ahmed M (1985). Plasma glucose and insulin profiles in normal subjects ingesting diets of varying carbohydrate, fat, and protein content. J Am Coll Nutr.

[11] Szendroedi J, Saxena A, Weber KS, Strassburger K, Herder C, Burkart V (2016). Cohort profile: the German Diabetes Study (GDS). Cardiovasc Diabetol.

[12] Weber KS, Straßburger K, Fritsch M, Bierwagen A, Koliaki C, Phielix E (2018). Meal-derived glucagon responses are related to lower hepatic phosphate concentrations in obesity and type 2 diabetes. Diabetes Metab.

[13] Liljequist D, Elfving B, Skavberg Roaldsen K (2019). Intraclass correlation—a discussion and demonstration of basic features. PLoS ONE.

[14] Berry SE, Valdes AM, Drew DA, Asnicar F, Mazidi M, Wolf J (2020). Human postprandial responses to food and potential for precision nutrition. Nat Med.

[15] Shafaeizadeh S, Muhardi L, Henry CJ, van de Heijning BJM, van der Beek EM (2018). Macronutrient composition and food form affect glucose and insulin responses in humans. Nutrients.

[16] Hirsch S, Barrera G, Leiva L, de la Maza MP, Bunout D (2013). Variability of glycemic and insulin response to a standard meal, within and between healthy subjects. Nutr Hosp.

[17] Vega-López S, Ausman LM, Griffith JL, Lichtenstein AH (2007). Interindividual variability and intra-individual reproducibility of glycemic index values for commercial white bread. Diabetes Care.

[18] Vrolix R, Mensink RP (2010). Variability of the glycemic response to single food products in healthy subjects. Contemp Clin Trials.

